# Ultrasensitive and Selective Detection of Glutathione by Ammonium Carbamate–Gold Platinum Nanoparticles-Based Electrochemical Sensor

**DOI:** 10.3390/life12081142

**Published:** 2022-07-28

**Authors:** Wei Wang, Jiandan Chen, Zhenzeng Zhou, Shanshan Zhan, Zhiyuan Xing, Hongying Liu, Linan Zhang

**Affiliations:** Department of Automation, Hangzhou Dianzi University, Hangzhou 310018, China; wangwei161225@163.com (W.W.); chenjiandan1107@163.com (J.C.); a707807373@163.com (Z.Z.); zsssay@126.com (S.Z.); yuanpipi2233@163.com (Z.X.)

**Keywords:** glutathione, electrochemical, sensor, Au@Pt nanoparticle, ammonium carbamate modified electrode

## Abstract

Determining the concentration of glutathione is crucial for developing workable medical diagnostic strategies. In this paper, we developed an electrochemical sensor by electrodepositing amino-based reactive groups and gold–platinum nanomaterials on the surface of glassy carbon electrode successively. The sensor was characterized by cyclic voltammetry (CV), field emission scanning electron microscope (FESEM), energy dispersive X-ray spectroscopy (EDX), and electrochemical impedance spectra (EIS). Results showed that Au@Pt nanoparticles with the size of 20–40 nm were presented on the surface of electrode. The sensor exhibits excellent electrocatalytic oxidation towards glutathione. Based on this, we devised an electrochemical biosensor for rapid and sensitive detection of glutathione. After optimizing experimental and operational conditions, a linear response for the concentration of GSH, in the range of 0.1–11 μmol/L, with low detection and quantification limits of 0.051 μM (S/N = 3), were obtained. The sensor also exhibits superior selectivity, reproducibility, low cost, as well as simple preparation and can be applied in human serum sample detection.

## 1. Introduction

Glutathione (GSH) is a tripeptide that plays an important role in living organisms [1,2,3]. It is well-known that the nominal level of GSH ranges from 0.5 to 10 mM in living cells and from 2–12 µM in physiological fluids for healthy individuals [4]. It has recently emerged as an important biomarker for cancers and various disease detection, as its concentration varies in the µM to mM range in biological cells and fluids [5]. Thus, to selectively and sensitively determine its concentration is crucial for developing workable medical diagnostic strategies and making early complications predictions. However, it is difficult to detect the concentration of glutathione due to the instability of glutathione in solution and its tendency to be oxidized to form disulfides. Thus far, scientists have proposed several different analytical methods for glutathione analysis, such as ultraviolet spectrophotometry [6], fluorescence spectrophotometry [7], mass spectrometry [8], high-performance liquid chromatography [9], flow injection analysis [10], and enzymatic [11] and electrochemical methods [12,13,14,15,16]. However, most of the aforesaid methods have some limitations, such as the necessity for complex and expensive instruments, the demand for skill-based operation, and time-consuming nature. Therefore, it is a pressing task to develop an affordable, simple, quick, and sensitive method for glutathione detection [17].

In these past years, the electrochemical method has received lavish attention [18,19] thanks to its high affordability, simple operation, quick response, high sensitivity, and outstanding selectivity. Previously reported modified electrodes for the detection of glutathione with electrochemical methods include graphene oxide (GO) [20], carbon materials (carbon nanotubes) [21,22,23], metal oxides [24,25,26], DNA probes [27,28,29], etc. Although these modified electrodes have great detection effects, the materials can be easily peeled off from the electrode surface due to the modification. In order to compensate for this shortcoming, gold–platinum nanomaterial is modified on the surface of the glassy carbon electrode (GCE) through electrochemical deposition. On the other hand, electrochemical disposal of GCE in ammonium carbamate solution by cyclic voltammetry method is an effective and highly repeatable strategy in electrode treatments, and appropriate solvation may trigger its electrochemical activity [30]. If we combined the modification of Au@Pt nanomaterials and electrochemical activation, it would significantly improve the electrochemical activity of the modified electrode. Thus, for the purpose of making the modified electrode more electrochemically active, the amino group was modified on the surface of the glassy carbon electrode to increase the active site before the gold–platinum nanomaterial was electrodeposited.

**Scheme 1:**
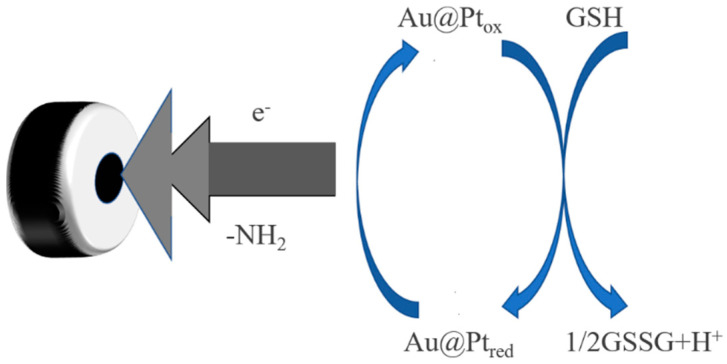
Scheme of Am-Au@Pt-based biosensor for glutathione detection.

In this paper, a composite chemical modification method was constructed by first electrodepositing an amino group in a solution of ammonium carbamate with the potentiostatic method [30], as shown in Scheme 1. On the basis of this, the gold–platinum nanomaterial modified glassy carbon electrode (Am-Au@Pt/GCE) with the cyclic voltammetry was fabricated by taking the advantage of amino group active and precious metal nanomaterials modification. The modified electrode was characterized by EIS, FESEM, and EDX as well as CV and exhibited excellent electrocatalytic oxidation towards glutathione. Subsequently, all experimental conditions were optimized. Last, glutathione was detected by the Am-Au@Pt/GCE biosensor, and an anti-interference test was also performed. Resulted showed that it can be applied in human serum sample detection.

## 2. Materials and Methods

Reagents: Chloroauric acid (HAuCl_4_, A.R.), chloroplatinic acid (H_2_PtCl_6_, A.R.), and glutathione (A.R.) were bought from Sangon Biotech Co., Ltd. (Shanghai, China). Ammonium carbamate (A.R.), sodium dihydrogen phosphate (A.R.), dipotassium phosphate (A.R.), L-cysteine (Cys), glutamine (Gln), glycine (Gly), glucose (Glu), ascorbic acid (AA), and absolute ethylalcohol (A.R.) were purchased from Aladdin Chemistry Co., Ltd. (Shanghai, China). Other chemicals used were of the analytical reagent grade. Additionally, 0.1 M phosphate buffer solution (PBS, pH = 7.4) was selected as the supporting electrolyte and was prepared with 0.1 M Na_2_HPO_4_ and NaH_2_PO_4_. All experiments were conducted at room temperature, and hyper-pure water was used.

Apparatus: The surface morphology and elemental composition were characterized with EDX (HORIBA EMAX X-ACT) and FESEM (JSM-6700F). Electrochemical experiments were conducted with a CHI660E electrochemical workstation (CH Instruments, Chenhua Co., Shanghai, China) with a standard three-electrode system, where a modified GCE was used as the working electrode, a platinum electrode was used as the counter electrode, and an Ag/AgCl electrode was used as the reference electrode.

Preparation of Am-Au@Pt/GCE: After being polished with Al_2_O_3_ powder (0.3 and 0.05 μm), GCE was ultrasonically cleaned with anhydrous ethanol and hyper-pure water for 3 min separately and then dried with N_2_. The sensor was prepared with two steps. First, GCE was placed in a 0.1 M ammonium carbamate solution and anodically polarized at +1.1 V for 1 h in a three-electrode system to obtain an Am/GCE [30]. Next, the Am/GCE was placed in a 0.5 M sodium chloride electrolyte containing 0.5 mM HAuCl_4_ and 0.167 mM H_2_PtCl_6_. The potential range was set at −1.3–0.8 V, and the operation was carried out at 10 mV/s for 3 sweep cycles to obtain Am-Au@Pt/GCE.

Electrochemical Experiments: To explore the electrochemical experiments of the prepared Am-Au@Pt/GCE, EIS and CV were employed. EIS was performed in a 0.1 M KCl solution containing 20 mM [Fe(CN)_6_]^3−/4−^ at an open circuit at 0.1–10^6^ Hz. CV was performed in a 0.1 M KCl solution including 20 mM [Fe(CN)_6_]^3−/4−^ under a voltage ranging from −0.2 to 0.6 V and at a scan rate of 100 mV/s.

The electrochemical detection of glutathione was realized by CV and the amperometric method (I-t). For CV, the operation was carried out at a voltage of 0–0.8 V and a scan rate of 100 mV/s. In I-t, for glutathione detection, the potential was 0.435 V.

## 3. Results

### 3.1. Characterization of Am-Au@Pt/GCE

**Figure 1:**
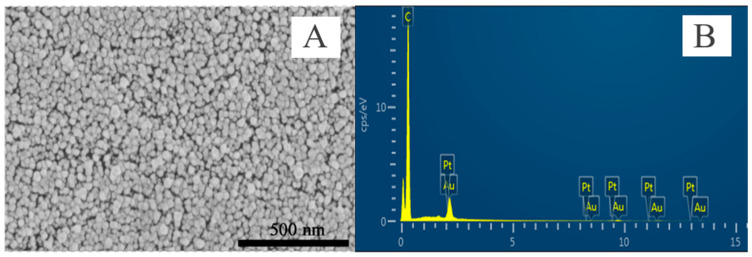
(**A**) FESEM of the as-prepared Am-Au@Pt/GCE; (**B**) EDS pattern of Am-Au@Pt/GCE.

The morphologies and composition of the Am-Au@Pt/GCE were first investigated by SEM and EDS, as shown in Figure 1. A dense pile of spherical Au@Pt nanomaterial was synthesized on the surface of the GCE, as shown in Figure 1A. They were neatly arranged with little cross-linking, and the sizes of the observed Au@Pt nanomaterials were within a range of 20–40 nm. Moreover, the EDS image of the Au@Pt prepared is shown in Figure 1B. It can be clearly seen from the image that Au and Pt are present on the surface of GCE. This indicates the successful preparation of Au@Pt nanomaterials on the surface of GCE. However, to understand the surface chemistry during glutathione interaction, a surface chemistry study with more detailed information will be conducted in the future.

Subsequently, the electrochemical properties of different modified electrodes were studied by EIS and CV. In EIS data, the semicircular portion corresponds to the high-frequency band of the electron transfer restriction process, while the linear part refers to the low-frequency band corresponding to the diffusion control process. The semicircle diameter of the high-frequency part is equal to the electron transfer resistance (R_et_). With the aid of the equivalent circuit diagram and the software fitting calculation, electron transfer resistances of GCE, Am-GCE, Am-Au/GCE, Am-Pt/GCE, and Am-Au@Pt/GCE were calculated to be 176, 21, 9, 10, and 5 Ω cm^−2^, respectively. It is obvious that the electron transfer resistance of Am-Au@Pt/GCE was the lowest. The presence of Am and Au@Pt can improve the conductivity of the electrode and accelerate the electron transfer between the electrode and the electrolyte. It also shows the successful modification of Am-Au@Pt/GCE. There may be three reasons for the resistance reduction: First, the regular atomic arrangement of gold–platinum nanomaterial is more beneficial to electron transmission. Second, the modified amino group on the surface of the GCE increases the specific surface area. Furthermore, it is possible that the synergistic effect of the amino group and the gold–platinum nanomaterial increases the electron transfer rate.

**Figure 2:**
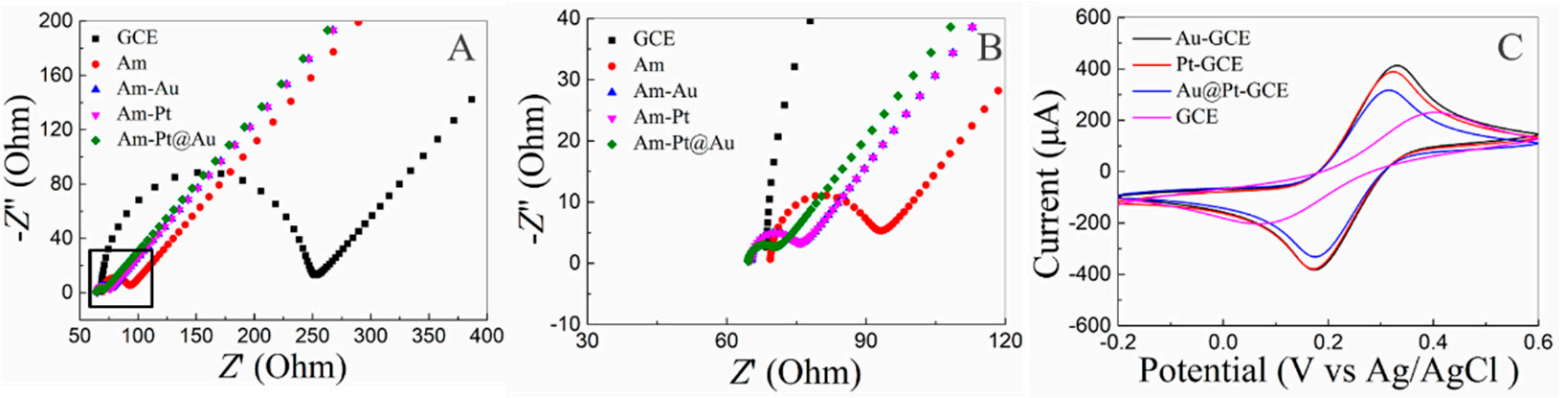
(**A**) Nyquist plots of EIS for GCE, Am-GCE, Am-Au/GCE, Am-Pt/GCE, and Am-Au@Pt/GCE in 0.1 M KCl solution that contains 20 mM K_3_Fe(CN)_6_/K_4_Fe(CN)_6_. The frequency ranged from 0.1 to 1.0 × 10^5^ Hz; (**B**) the enlarged view of (**A**); (**C**) CVs of GCE, Am-Au/GCE, Am-Pt/GCE, and Am-Au@Pt/GCE in 0.1 M KCl solution containing 20 mM K_3_Fe(CN)_6_/K_4_Fe(CN)_6_), with a scan rate was 100 mV s^−1^.

Furthermore, the electrochemical performance of various modified electrodes was further explored with cyclic voltammetry using 20 mM [Fe(CN)_6_]^3−/4−^ as a redox probe, as shown in Figure 2C. The oxidation peak currents of Am-Au/GCE, Am-Pt/GCE, and Am-Au@Pt/GCE are significantly higher than that of bare GCE. This indicates that the modified electrode can accelerate the electron transfer between the electrode and the probe. Plus, the peak potential differences (ΔE_p_) of the modified electrodes were compared. The oxidation peak potential differences (ΔE_p_) of Am-Au/GCE, Am-Pt/GCE, and Am-Au@Pt/GCE were calculated to be 157 mV, 152 mV, and 139 mV, respectively, indicating that Am-Au@Pt/GCE can enhance the electron transfer between electrode and [Fe(CN)_6_]^3−/4−^.

### 3.2. Electrocatalytic Activities of Glutathione on the Modified Electrode

**Figure 3:**
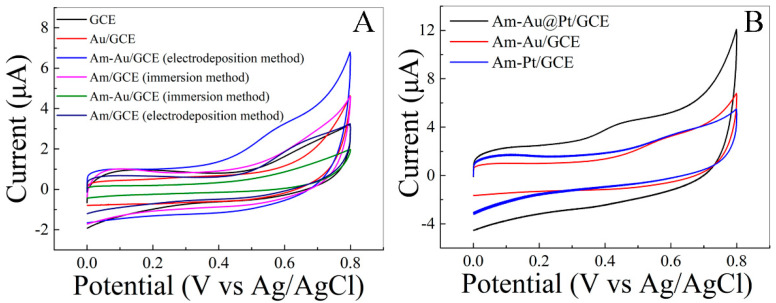
(**A**) CVs of GCE, Au/GCE, Am-Au/GCE (electrodeposition method), Am/GCE (immersion method), Am-Au/GCE (immersion method), and Am/GCE (electrodeposition method) in PBS (pH 7, 0.1 M) containing 100 μM glutathione with a voltage of 0–0.8 V and a scan rate of 100 mV/s; (**B**) CVs of Am-Au/GCE, Am@Pt/GCE, and Am-Au@Pt/GCE in PBS (pH 7, 0.1 M) containing 100 μM glutathione from 0 to 0.8 V and at a scan rate of 100 mV/s.

In a bid to verify the feasibility of this method, the electrochemical behavior of glutathione on different modified electrodes were investigated, as shown in Figure 3A. No obvious oxidation peak could be obtained at bare GCE and Am/GCE prepared with the immersion method. It shows that the way of immersing the modified amino group has no catalytic effect on glutathione detection. However, a distinct oxidation peak was observed for Am-GCE prepared with the electrodeposition method. It shows that the electrodeposition method is an effective strategy, and the amino group modified by electrodeposition has a good electrocatalytic effect on glutathione detection. For further exploration, the peaks of Au/GCE and Am-Au/GCE prepared with the immersion method and Am-Au/GCE prepared with the electrodepositing method were compared. The results show that the modification of amino affects gold deposition. The peak current of Am-Au/GCE prepared with the electrodepositing method was higher than those of others. There is a synergistic effect between the amino group modified by electrodeposition and the deposited gold, which is probably due to the fact that the lone pair electrons on electrodeposition modified amino are more active, which is conducive to the formation of covalent bonds with gold, and thus advantageously increase the electron transfer rate. Lastly, the electrocatalytic activity of glutathione on the Am-Au/GCE, Am-Pt/GCE, and Am-Au@Pt/GCE was explored. Figure 3B clearly shows a negative shift in oxidation peak potential and the greatly increased oxidation peak current. This indicates that Am-Au@Pt/GCE has better electrocatalytic effects on glutathione, and gold–platinum nanomaterial increases the rate of electron transfer and has better electrocatalytic selectivity for glutathione. In summary, Am-Au@Pt/GCE is used as an electrochemical sensor for glutathione detection due to a high surface area for glutathione adsorption of glutathione and high catalytic activity and electronic conductivity of the nanostructured material. The reaction mechanisms catalyzed by the nanomaterial are described as follows [31]:

(1) $$M_{\mathrm{ox}}+e^{-}\to M_{\mathrm{red}}$$

(2) $$\mathrm{GSH}\to \frac{1}{2}\;{\mathrm{GSSG}+H}^{+}+e^{-}$$

### 3.3. Optimization of Electrodepositing Conditions

**Figure 4:**
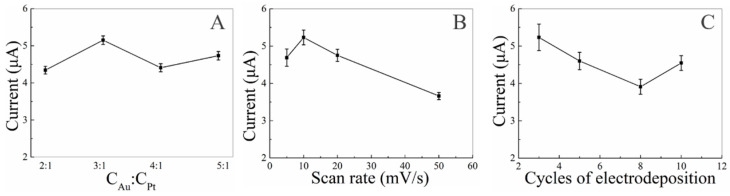
Effects of the concentration ratio of chloroplatinic acid to chloroauric acid (**A**), the sweep rate (**B**), and the number of electrodeposition scanning cycles (**C**) on the oxidation peak current response of Am-Au@Pt/GCE towards glutathione.

For the best electrochemical performance of the modified electrode, the preparation conditions were optimized. Firstly, we studied the ratio of chloroauric acid to chloroplatinic acid in the electrodeposition solution and the electrocatalytic properties of electrodes prepared in the electrodeposition solution with different concentration ratios. As shown in Figure 4A, the highest peak current was observed at the chloroauric acid to chloroplatinic acid ratio of 3:1, indicating the modified electrode possesses the highest catalytic values towards glutathione. Subsequently, the scanning rate of the modified electrode in the electrodeposited gold–platinum nanomaterial was optimized because the particle size of the material deposited on the electrode surface affected the electrocatalytic performance of the modified electrode, as shown in Figure 4B. It can be seen that the current response first increased and then decreased when the scan rate went up and reached the maximum when the scan rate was 10 mV/s. The reason may be that the particle size of the material deposited on the electrode surface affected the electrocatalytic performance of the modified electrode. Small Au@Pt nanoparticles size resulted in a low electron transfer speed, while the catalytic behavior was significantly reduced when the particle size was too large. Thus, the deposition scan rate of 10 mV/s was chosen. The number of scanning turns affects the thickness of the material deposited on the electrode surface and the electrocatalytic performance of the modified electrode. Thus, the number of scanning circles for electrodeposition was optimized finally. As shown in Figure 4C, the modified electrode had the best electrocatalytic performance when there were three cycles. In summary, the optimal electrodeposition conditions were determined as follows: the concentration ratio of chloroplatinic acid to chloroauric acid is 1:3, the sweep rate of electrodeposition is 10 mV/s, and the number of scanning cycles of electrodeposition is three.

### 3.4. Effect of pH

**Figure 5:**
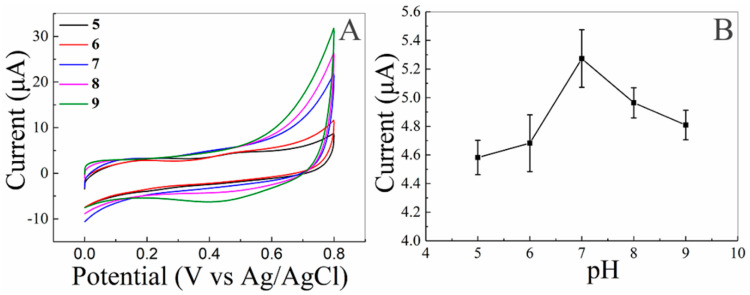
(**A**) CVs of 100 μM glutathione detected on Am-Au@Pt/GCE at different pH values (pH = 5, 6, 7, 8, 9) in 0.1 M PBS; (**B**) line diagram of oxidation peak current at different pH in (**A**).

It is well-known that in electrochemical methods, pH of the electrolyte is very important, and the electrocatalytic properties and pH of the modified electrode are also inextricably linked. Therefore, the detection of glutathione with Am-Au@Pt/GCE at different pH values was investigated. Figure 5A shows that the hydrogen evolution reaction of the modified electrode gradually increased with the increase in pH value. In Figure 5B, the oxidation peak current of the glutathione gradually increased and then decreased with the increase in pH value. The maximum value was obtained at a pH of 7. Therefore, a pH of 7 was chosen as the optimal parameter for the following experiment.

### 3.5. Quantitative Detection of Glutathione

**Figure 6:**
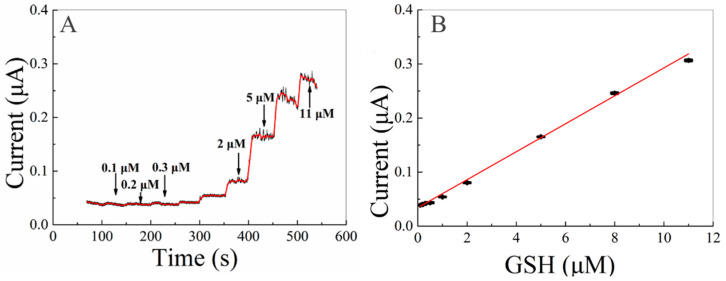
(**A**) I-t response of Am-Au@Pt/GCE in the presence of 0.1–11 μM glutathione at a working potential of 0.435 V; (**B**) calibration plots of response current of glutathione vs. the concentration of glutathione.

Through the above exploration, the concentration of glutathione was determined by I-t at a working voltage of 0.435 V, and a linear relationship between the peak current and the concentration was established. As shown in Figure 6A, glutathione with a certain concentration was added per 50 s. It can be seen that the current response gradually increased with the increase in the concentration of glutathione. A good linear correlation in the range of 0.1–11 μM was observed. The regression equation is Ipa (μA) = 0.034 + 0.026C (μM), R^2^ = 0.995. The detection limit was calculated to be 0.051 μM (S/N = 3) using 10 sets of parallel blanks. Compared with some of the previously described modified electrodes (Table 1), our sensor has a low detection limit and a wide detection range. Furthermore, we compared our proposed method with other reported GSH detection methods in Table 2. Our method is one of the most effective methods reported so far.

### 3.6. Interference and Repeatability Studies

**Figure 7:**
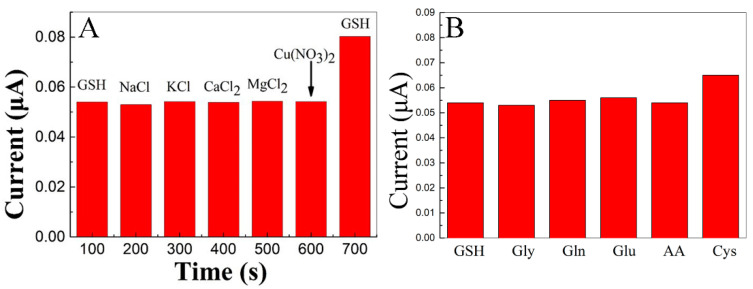
(**A**) Comparing histograms of the amperometric response of the Am-Au@Pt/GCE with the accumulated addition of 1 μM glutathione, 100 μM NaCl, 100 μM KCl, 100 μM CaCl_2_, 100 μM MgCl_2_, 100 μM Cu(NO_3_)_2_, and 1 μM glutathione in 0.1 M PBS (pH 7) at a working potential of 0.435 V. (**B**) Selectivity of the sensor for GSH determination against biological interferences, including glycine, glutamate, glucose, cysteine, and ascorbic acid, under the best experimental condition.

To verify the specificity of Am-Au@Pt/GCE for glutathione detection, an anti-interference experiment was performed. Figure 7A shows the current response of the Am-Au@Pt/GCE with the accumulated addition of 1 μM glutathione, 100 μM NaCl, 100 μM KCl, 100 μM CaCl_2_, 100 μM MgCl_2_, 100 μM Cu(NO_3_)_2_, and 1 μM glutathione in 0.1 M PBS (pH 7) at a working potential of 0.435 V. It can be seen from the figure that the current response is hardly affected after the addition of interfering ions. The current response suddenly increased when 1 μM glutathione was added at 700 s, indicating that Am-Au@Pt/GCE is very selective for detecting glutathione. Furthermore, several similar amino acids (glycine and glutamate), glucose, cysteine, and ascorbic acid were also tested. As shown in Figure 7B, the response to these interferences other than Cys is almost negligible. The reason may be the high binding affinity between the thiol and Au@Pt nanoparticles through forming Au-S bonds. However, the concentration of GSH is remarkably higher than that of Cys in biological systems. Thus, the selectivity of the proposed electrochemical biosensor is acceptable for biological systems.

For the purpose of testing the repeatability of Am-Au@Pt/GCE, the same electrode was used to detect 5 μM glutathione six times. Its relative standard deviation is 3.7%, which indicates that the repeatability of the electrode is also fairly good. Furthermore, the repeatability was also confirmed by *t*-tests. The two-tailed *t*-test carried on Warburg coefficient values (*n* = 3) were at a 95% confidence interval (*p* < 0.5) for two different concentrations (5 and 10 μM glutathione). The error value was within ±10%, indicating an acceptable variation.

### 3.7. Determination of GSH in Human Serum Samples

Human serum samples obtained from the Hospital of Hangzhou Dianzi University were used to evaluate the application of the proposed biosensor. The standard addition method was used to detect the content of GSH. From Table 3, it can be seen that the recovery of GSH in human serum was between 95% and 107%, indicating that the proposed biosensor possessed good reliability in practical applications.

## 4. Conclusions

In summary, a composite chemical modification method for modifying the amino group on the surface of glassy carbon electrode in an ammonium carbamate solution with a potentiostatic method was introduced. Then, the nano-gold–platinum nanomaterial was modified by cyclic voltammetry to obtain Am-Au@Pt/GCE. The electrochemical biosensor proposed in this paper has excellent electrocatalytic oxidation effects on glutathione. Therefore, a new type of electrochemical biosensor for rapid and sensitive glutathione detection was designed and built. The preparation conditions of the sensor were optimized and characterized by EIS, FESEM, EDX, and CV. Under the best experimental condition, the detection range of glutathione is 0.1–11 μM, and the detection limit is 0.051 μM (S/N = 3). Finally, it was used to detect GSH in human serum sample. In the future, this biosensor is likely to be used for the detection of other small biological molecules as well as batteries and in other fields thanks to its excellent electrochemical performance and great stability.

## Figures and Tables

**Table 1:** Comparison of varying electrodes for determination of glutathione.

Type of Electrode	Detection Limit (μM)	Liner Range (μM)	Ref
Nano-TiO_2_/ferrocene carboxylic acid	0.098	0.1–12	[32]
Au@MnO_2_	0.11	0.33–16	[33]
Au-Ag bimetallic nanoclusters	0.2	1–100	[34]
PtNPs@MnO_2_	0.05	0.2–11	[35]
Am-Au@Pt/GCE	0.051	0.1–11	This work

**Table 2:** Comparison of different methods for determination of glutathione.

Methods	Detection Limit (μM)	Liner Range (μM)	Ref
High-performance liquid chromatography (HPLC)	0.5	0.75–10	[36]
Fluorescence	0.06	0–10	[37]
ECL	0.05	0.1–1.0	[38]
Photoelectrochemical	0.01	0.01–10	[39]
Electrochemical	0.051	0.1–11	This work

**Table 3:** The application of the proposed biosensor for the determination of human serum spiked with different concentrations of GSH.

Sample	Spiked (μM)	Found (μM)	Recovery (%)	RSD (%)
Human Serum	0.5	0.52	104	5.9
	1	0.95	95	3.2
	5	5.36	107	4.6

## Data Availability

Not applicable.
